# Bioinformatics Education—Perspectives and Challenges

**DOI:** 10.1371/journal.pcbi.0010052

**Published:** 2005-11-25

**Authors:** Shoba Ranganathan

Education in bioinformatics has undergone a sea change, from informal workshops and training courses to structured certificate, diploma, and degree programs—spanning casual self-enriching courses all the way to doctorate programs. The evolution of curriculum, instructional methodologies, and initiatives supporting the dissemination of bioinformatics is presented here.

Building on the early applications of informatics (computer science) to the field of biology, bioinformatics research entails input from the diverse disciplines of mathematics and statistics, physics and chemistry, and medicine and pharmacology. Providing education in bioinformatics is challenging from this multidisciplinary perspective, and represents short- and long-term efforts directed at casual and dedicated learners in academic and industrial environments. This is an NP-hard problem.

Training in bioinformatics remains the oldest and most important rapid induction approach to learning bioinformatics skills. Both formal (short-term courses) and informal training (on-demand “how-to” procedures) have remained the mainstay of on-the-job programs.

After almost a decade of short-term training, and retraining students, faculty, and scientists in discrete aspects of bioinformatics, the impetus to formalize bioinformatics education came in 1998 from Russ Altman [1], with a wish list of topics for an ideal bioinformatics educational program at the masters and PhD levels. Given the multidisciplinary nature of bioinformatics and the need for designing cross-faculty courses, by 2001 only a handful of universities had successfully commenced formal education in bioinformatics, with others waiting and watching.

The Workshop on Education in Bioinformatics (WEB) 2001 (http://surya.bic.nus.edu.sg/web01/) was launched at the 2001 International Conference on Intelligent Systems for Molecular Biology (ISMB) as a satellite meeting, to provide for the first time a forum for bioinformatics educators to meet, discuss, and exchange ideas and suggestions. WEB addresses fundamental educational and pedagogical issues to determine the nature, extent, and content of, and delivery tools available for, bioinformatics degree and training programs, and to provide focus points and suggestions for improvement of nascent degree programs.

WEB has served as the single annual meeting for education in bioinformatics, with plenary, oral, and poster presentations. WEB has, over the past four years, witnessed the evolution of bioinformatics education through talks and posters. Typically, presentations have sequentially progressed from a masters degree to a PhD [2,3] or from a minor to a complete program [4]. Several WEB attendees themselves started educational programs [5] and are now active members of the ISCB Education Committee, where a list of currently available educational programs is maintained [6]. The new educational initiatives showcased include the S* Life Science Informatics Alliance in WEB 2001 and the Worldwide Universities Network in WEB 2002. Special sessions on industry needs (WEB 2002) and pedagogy (WEB 2003 and WEB 2004) have also been included in the program to provide perspective and depth.

Bioinformatics education became mainstream with the ISMB 2005 conference, featuring a joint education workshop integrating WEB with the efforts of the ISCB's Education Committee (referred to as WEB 2005 here) on the opening day of the conference.

How well do the new graduates from these educational programs fit the jobs available to them? How many areas should a bioinformatics graduate be an expert in? Apart from a deep understanding of algorithms, programming, and life sciences, solving problems in genomics, proteomics, and/or medical informatics appears to be the current requirement. With the blurring boundaries between bioinformatics and new areas of endeavor such as forensics and biodiversity/ecoinformatics, where should we, as educators, draw the line? With industry's annual fluctuating demands for specific in-depth knowledge, how can we create a bioinformatics world?

Some of these issues will be discussed in the 2006 WEB meeting. But here are four take home messages from the earlier workshops. First, with the growing demand for computational biologists, there is a persistent and continuing demand for bioinformatics education at all levels—formal and informal, face-to-face and distance learning, and short-term training and rigorous long-term academic programs. Clearly, “one size does not fit all” [7], since the trend is to develop new and innovative educational programs addressing niche areas within bioinformatics. Second, there is still no single bioinformatics education program that can satisfy today's wish list of essential ingredients [8,9]. But by enabling the student cohort in self-learning, with a problem-based approach [10], the ontology of education (“to draw forth”) is realized. Third, percolation of basic bioinformatics down to the undergraduate level, especially for all life science majors [11] and optionally for physical and computer science majors [12] would develop the multidisciplinary skills required for bioinformatics. Fourth, “faster, further, and more” summarizes the components that would go into curricula of the future. ISCB members, educators, and bioinformatics professionals are encouraged to participate in WEB, and contribute their valuable ideas and expertise to this bioinformatics educational initiative. 

## Abbreviations

ISMB: Intelligent Systems for Molecular Biology
WEB: Workshop on Education in Bioinformatics
